# Public Mental Health in internationaler Perspektive: vom Shifting the Curve zur Inklusion vulnerabler Populationen

**DOI:** 10.1007/s00103-023-03673-9

**Published:** 2023-02-27

**Authors:** Ulrich Reininghaus, Christian Rauschenberg, Anita Schick, Jessica A. Hartmann

**Affiliations:** 1 Abteilung Public Mental Health, Zentralinstitut für Seelische Gesundheit, Medizinische Fakultät Mannheim, Universität Heidelberg, Mannheim, Deutschland; 2 Centre for Epidemiology and Public Health, Health Service and Population Research Department, Institute of Psychiatry, Psychology & Neuroscience, King’s College London, London, Großbritannien; 3 ESRC Centre for Society and Mental Health, King’s College London, London, Großbritannien; 4 Orygen, the National Centre of Excellence in Youth Mental Health, Parkville, Australien

**Keywords:** Öffentliche psychische Gesundheit, Populationsstrategie, Psychische Gesundheit der Bevölkerung, Vulnerable Populationen, Public mental health, Population strategy, Population mental health, Vulnerable populations

## Abstract

In den letzten Jahren haben sich die Anstrengungen im Bereich der Public Mental Health intensiviert, die psychische Gesundheit und Gesundheitskompetenz auf Bevölkerungsebene zu stärken sowie Fortschritte in der Prävention und Versorgung von psychischen Erkrankungen zu erzielen. Der vorliegende Beitrag gibt einen Überblick über derzeitige Konzeptualisierungen von Indikatoren und Determinanten der Public Mental Health sowie von populationsbasierten Interventionsstrategien aus internationaler Perspektive. Derzeitige konzeptionelle und methodische Herausforderungen von sogenannten Hochrisikostrategien, Populationsstrategien und dem vulnerablen Populationsansatz werden kritisch diskutiert. Zukünftige Anstrengungen in Politik, Forschung und Praxis sollten fundamentale Ursachen sozialer und gesundheitlicher Ungleichheiten unter Einbezug aller gesellschaftlichen Handlungsfelder stärker in den Blick nehmen, um einen Beitrag zur Verbesserung der populationsbasierten Gesundheit zu leisten.

## Abstract (en)

In recent years, efforts in the field of public mental health have increased that seek to promote mental health and mental health literacy at population level and yield advances in the prevention, treatment and care of mental health conditions. This paper provides an overview of contemporary conceptualisations of indicators and determinants of public mental health as well as population-based intervention strategies from an international perspective. Current conceptual and methodological challenges of so-called high-risk, whole-population and vulnerable population strategies are critically discussed. Future efforts in research, policy and practice need to address fundamental causes of social and health inequalities, drawing on all societal fields, to contribute to improving population mental health.

## Einleitung

In den vergangenen Jahren hat das noch relativ junge Fach Public Mental Health in der internationalen Forschung zunehmend an Bedeutung gewonnen. Derzeitige Konzeptualisierungen der Public Mental Health im internationalen Raum fokussieren auf Indikatoren, Determinanten, Förderung bzw. Schutz der psychischen Gesundheit, Verbesserung der Gesundheitskompetenz, die Reduktion von Stigmata sowie auf Prävention von psychischen Störungen und deren Versorgung [1–4]. Häufig standen dabei bislang entweder die Anwendung von vorwiegend an somatischen Erkrankungen entwickelten Ansätzen der Public Health auf den Bereich der psychischen Gesundheit und Erkrankung im Vordergrund [4, 5] oder deren populationsbasierte, epidemiologische oder systemische Betrachtung aus psychiatrischer Perspektive [6]. Ein Grund dafür, dass die Public Mental Health lange Zeit nicht per se im Mittelpunkt der Betrachtung stand, dürfte darin liegen, dass im Fach Public Health über viele Jahre somatische Erkrankungen im Fokus der Aufmerksamkeit standen [5], während sich die Psychiatrie – wie Geoffrey Rose bereits in den 1990er-Jahren feststellte – nicht der Existenz und Wichtigkeit des Gesundheitszustandes von ganzen Populationen bewusst zu sein schien, sondern sich vielmehr, ihrer primären Aufgabe folgend, vorwiegend mit der Subpopulation von Menschen mit diagnostizierter psychischer Erkrankung befasste [7, 8].

Dabei wird schnell deutlich, dass es eine besondere Herausforderung der Public Mental Health ist, sie in ihrer Interdisziplinarität von Public Health, psychiatrischer Epidemiologie, psychischer Gesundheitsförderung, Prävention und Versorgungsforschung über das gesamte Spektrum von psychischer Gesundheit und Erkrankung hinweg abzubilden, um ihr innovatives Potenzial vollständig auszuschöpfen [3]. Diese Interdisziplinarität ist der Public Mental Health insofern inhärent, als dass sie sich mit dem gesamten – kontinuierlich verteilten – Spektrum von psychischer Gesundheit und Erkrankung befasst. Sie muss ein zentraler Gegenstand von Wissenschaft und Praxis der Public Mental Health sein, um eine entscheidende Rolle für die wissenschaftliche Untersuchung und evidenzbasierte Verbesserung der psychischen Gesundheit für die gesamte Bevölkerung spielen zu können [3].

Obwohl auch im internationalen Raum Psychiatrie und Public Health zuweilen noch in ihren disziplinären Perspektiven verharren (wie bspw. im Vereinigten Königreich in der Debatte um den Jahresbericht des Chief Medical Officer 2013 „Public Mental Health Priorities: Investing in the Evidence“ deutlich wurde [1, 9]) und die Beantwortung fundamentaler konzeptioneller und methodischer Probleme noch aussteht [3], so hat die Public Mental Health in den letzten Jahren dennoch an Momentum gewonnen. Die Gründe dafür sind vielfältig: Gesellschaftlich hat das Thema der psychischen Gesundheit – zuletzt katalysiert durch die psychosozialen Folgen der COVID-19-Pandemie [10, 11] – mehr Aufmerksamkeit erfahren [3], auf politischer Ebene haben Weltgesundheitsorganisation (WHO) und Regierungen [2, 12–16] erkannt, dass Forschung und Praxis der Public Mental Health gestärkt werden müssen, und in der internationalen Wissenschaftsgemeinde ist Inter- und Transdisziplinarität nicht mehr die Ausnahme, sondern die Regel.

Der vorliegende Beitrag gibt einen Überblick über derzeitige Konzeptualisierungen von Indikatoren und Determinanten der Public Mental Health sowie von populationsbasierten Interventionsstrategien aus internationaler Perspektive. Aktuelle konzeptionelle und methodische Herausforderungen von Hochrisikostrategien, Populationsstrategien und dem vulnerablen Populationsansatz werden dargestellt und kritisch beleuchtet.

## Konzeptualisierungen von Indikatoren und Determinanten der Public Mental Health

Indikatoren und Determinanten der Public Mental Health haben u. a. Niederschlag in Systemen für Surveillance, Monitoring und Assessment gefunden, wie sie von der WHO gefordert [17] und bereits in einigen Ländern mit unterschiedlichen Schwerpunkten etabliert worden sind [2, 18]. So nahmen am globalen Reporting von Public-Mental-Health-Indikatoren im Rahmen des Projekts „Mental Health Atlas“, das im Jahr 2001 von der WHO initiiert wurde, im Jahr 2017 177 der 194 Mitgliedstaaten zumindest teilweise teil [16]. Dabei verfügten 62 % der Mitgliedstaaten über eine ausreichende Datengrundlage, um über ein Set von 5 ausgewählten Indikatoren in den Bereichen Politik, Gesetzgebung, Versorgung, Personal und Gesundheitsförderung/Prävention berichten zu können [16].

In Kanada stehen bspw. Risiko- und Schutzfaktoren [19], positive psychische Gesundheit [20], diagnostizierte psychische Erkrankungen [19] und Suizidalität [21] im Mittelpunkt der nationalen Public Health Surveillance. Ausgehend vom 2‑Kontinua-Modell (in dem psychische *Gesundheit* und psychische *Erkrankung* als 2 distinkte Kontinua repräsentiert sind, die jedoch miteinander in Beziehung stehen [22, 23]) werden in der Schweiz positive psychische Gesundheit und psychische Beschwerden separat betrachtet und zusammen mit Risiko- und Schutzfaktoren, Suizidalität und Inanspruchnahme der Gesundheitsversorgung durch das Schweizerische Gesundheitsobservatorium dokumentiert und berichtet [15]. In Australien wird für das Monitoring der psychischen Gesundheit bzw. des psychischen Wohlbefindens der australischen Bevölkerung sowie der Leistung der psychischen Gesundheitsversorgung eine Zusammenstellung von unterschiedlichen psychosozialen Indikatoren (z. B. psychische Belastung, Prävalenz psychischer Erkrankungen, soziale Teilhabe, Suizidrate) und Inanspruchnahmedaten (z. B. Rehospitalisierungsrate, Zwangseinweisungen, Versorgungszugang) verwendet [13]. In Schottland werden Indikatoren von positiver psychischer Gesundheit und psychische Gesundheitsprobleme gemeinsam mit kontextuellen Risiko- und Schutzfaktoren auf individueller, kommunaler und struktureller bzw. politischer Ebene erhoben [24, 25]. Zudem hat die School for Public Health Research (SPHR) im Vereinigten Königreich kürzlich eine umfassende Konzeptualisierung veröffentlicht, in der in einem intensiven Konsultationsprozess mit unterschiedlichen Stakeholdern 55 Determinanten der Public Mental Health priorisiert und anhand von 4 Ebenen (Individuum, Familie, Gemeinde, strukturell) gruppiert wurden [2].

Dabei verbindet die meisten dieser Ansätze, dass sie sich aus unterschiedlichen Datenquellen speisen, die zur Datenerhebung wiederum Messinstrumente und -methoden verwenden, die für sich genommen in unterschiedlichem Ausmaß empirisch validiert sind. Insgesamt bleibt die Evidenz hinsichtlich einer umfassenden empirischen Validierung von Indikatoren gemeinsam mit Determinanten derzeitiger Konzeptualisierungen begrenzt. Zudem werden wichtige (Weiter‑)Entwicklungen in der Operationalisierung des gesamten Spektrums von psychischer Gesundheit und Erkrankung nicht ausreichend berücksichtigt.

So erfahren aktuelle Fortschritte im Bereich von kontinuierlichen und dimensionalen Ansätzen der internationalen Forschung bislang nur begrenzte Aufmerksamkeit. Dies ist bspw. der Fall im Rahmen der *Kontinuumshypothese *[26], die psychisches Erleben entlang eines Kontinuums von psychischer Gesundheit und Erkrankung verteilt betrachtet, oder auch in der *Hierarchical Taxonomy of Psychopathology* (HiTOP; [27]), in deren Mittelpunkt dimensionale Modelle der Psychopathologie zur Klassifikation von psychischen Erkrankungen stehen. Diese Modelle sind erforderlich, um Maße der zentralen Tendenz (z. B. Mittelwert) und andere – für Public Mental Health – wichtige epidemiologische Parameter (z. B. Inzidenz anhand empirischer Schwellenwerte [27]) auf Populationsebene valide bestimmen zu können [3]. Dabei werden die Ansätze durch die derzeitige (metaanalytische) Evidenz in der Forschung zur Klassifikation von psychiatrischen Erkrankungen unterstützt [28–33].

Zudem sollten die jeweiligen Populationsparameter im Kontext des *Clinical Staging Model* von psychischen Erkrankungen betrachtet werden [34], das – ähnlich der Staging-Modelle zu Tumorerkrankungen in der somatischen Medizin – zwischen unterschiedlichen Risiko- und Erkrankungsstadien (von frühen Risikostadien bis hin zu späten, behandlungsresistenten Stadien psychischer Erkrankungen) unterscheidet [3]. Während diese bislang primär im Kontext der Früherkennung von psychotischen Störungen entwickelt und untersucht wurden, beinhalten die neuesten Modelle mittlerweile transdiagnostische klinische Risikostadien von schweren psychischen Erkrankungen [35–38], die bspw. in der Version von Hartmann et al. [35] zwischen einer nichtspezifischen Belastung (Stadium 1a), breitem Hochrisikosyndrom (Broad Clinical High At-Risk Mental State – CHARMS, Stadium 1b) und der ersten Episode einer schweren psychischen Erkrankung (Stadium 2) differenzieren.

Diese bislang separaten Silos bedürfen zwingend einer integrierten Untersuchung, um Risikostadien im Kontext von populationsbasierten Ansätzen zu validieren sowie anhand von dimensionalen Skalen evidenzbasierte Schwellenwerte für unterschiedlich gestufte Erkrankungsrisiken zu generieren.

Eine weitere eng damit verbundene zentrale Herausforderung ist, dass sich die meisten Modelle der psychischen Gesundheit primär auf Kategorien oder Dimensionen der Psycho*pathologie* konzentrieren, obwohl spätestens seit den Definitionen der WHO immer wieder betont wird, dass psychische Gesundheit mehr als die Abwesenheit von Psychopathologie ist [17]. Dabei sind die Erfassung von positiver psychischer Gesundheit, psychischem Wohlbefinden und Genesung (Recovery) sowie die Betrachtung von Schutzfaktoren zentral für die Aufgaben und Funktionen im Bereich von Gesundheitsförderung, Gesundheitsschutz, Prävention und psychiatrischer Versorgung. Allerdings wurden diese bislang nicht entlang des Spektrums zusammen mit klinischen (Risiko‑)Stadien, psychopathologischen Dimensionen und Inzidenzen von psychischen Erkrankungen kalibriert. Es fehlt also kurzgefasst ein empirisch validiertes und umfassend kalibriertes Klassifikationssystem der populationsbasierten psychischen Gesundheit [3].

## Populationsbasierte Strategien der Public Mental Health

Den bis hierhin dargestellten Konzeptualisierungen entsprechend hat die Public Mental Health auch hinsichtlich der Gesundheitsversorgung das gesamte Spektrum der psychischen Gesundheit im Blick und umfasst somit:

1. Förderung bzw. Schutz der psychischen Gesundheit (u. a. in gesellschaftlichen Krisen),
2. Förderung der Kompetenz im Bereich der psychischen Gesundheit und damit eng verbunden die Reduktion von Stigmata,
3. Verhaltens- und Verhältnisprävention von psychischen Erkrankungen und
4. Versorgung von Menschen mit psychischer Erkrankung [3].

Wie in anderen Bereichen der Public Health kommt auch in der Public Mental Health populationsbasierten Interventionen eine besondere Bedeutung zu, deren Anliegen es ist, die psychische Gesundheit der gesamten Bevölkerung zu verbessern [39]. Dabei lassen sich in der internationalen Literatur mindestens 3 Arten von populationsbasierten Interventionen unterscheiden:

1. Interventionen, deren Ziel das Segment der Population mit dem höchsten Risiko ist (Hochrisikostrategie),
2. Interventionen, deren Ziel die Verschiebung der Verteilung (Shifting the Curve) von Risiken und Gesundheit der gesamten Population ist (Populationsstrategie),
3. Interventionen, deren Ziel vulnerable (Sub)Populationen sind, die aufgrund geteilter sozialer Charakteristiken bzw. ihrer Position in den sozialen Schichten ein höheres Risiko einer Risikoexposition aufweisen und deshalb systematische soziale und gesundheitliche Ungleichheiten erfahren, die es zu reduzieren gilt (vulnerabler Populationsansatz; [39]).

### Von Hochrisikostrategien für schwere psychische Erkrankungen zum Konzept der Frühintervention

Ein wichtiger Meilenstein in der Entwicklung von Hochrisikostrategien und in der Public-Health-Politik insgesamt stellt ein Bericht [40] aus dem Jahr 1974 dar, in dem der kanadische Gesundheits- und Sozialminister Marc Lalonde vorschlug, sich bei populationsbasierten Interventionen auf dasjenige Segment der Population zu konzentrieren, welches das höchste Erkrankungsrisiko aufweist. In der Psychiatrie erfuhr diese Strategie erst einige Jahre später ihre Anwendung im Bereich der Früherkennung und Frühintervention von psychotischen Erkrankungen, die in engem Zusammenhang mit den bereits erwähnten klinischen Risikostadien zu sehen sind.

Beginnend in den 1990er-Jahren stellten die Entwicklung und Implementierung von Early Intervention (EI) Services für psychotische Erkrankungen den Beginn eines globalen Wandels innerhalb der Psychiatrie dar: von einem Fokus fast ausschließlich auf die Behandlung chronischer, persistierender Krankheit hin zu einem Modell zur Erkennung und Behandlung von frühen, teils unter dem Schwellenwert für eine erste Diagnose liegenden Stadien, welches später als die „Youth Mental Health Reform“ bezeichnet wurde [41–43]. Dabei wurzelt das Konzept der Frühintervention in der Beobachtung, dass die große Mehrheit der psychischen Erkrankungen (ca. 75 %) während einer der kritischsten und sensitivsten Lebensphasen eines Menschen auftreten, nämlich vor dem 25. Lebensjahr [44]. Infolgedessen können signifikante Meilensteine in der persönlichen Entwicklung nicht erreicht werden. Die damit verbundenen sozioökonomischen Auswirkungen psychischer Erkrankungen übertreffen diejenigen von kardiovaskulären Erkrankungen und Krebs [45, 46].

Das Frühinterventionsmodell und die Hochrisikostrategie zeigten, dass frühe Identifizierung und zeitgerechte Behandlung die Prognose verbessern können [34] und eine kosteneffektive Strategie darstellen [47, 48]. Obwohl junge Menschen den größten Bedarf an psychischer Gesundheitsversorgung haben und das größte Potenzial für einen Behandlungserfolg aufweisen, ist der Zugang zu medizinischer Versorgung weltweit für junge Menschen häufig erschwert und mit langen Wartezeiten verbunden [43]. Eine vielfach kritisierte Spaltung zwischen Pädiatrie, Kinder- und Jugend- und Erwachsenenpsychiatrie zum 18. Lebensjahr kann zu einem Versorgungsengpass und gescheiterten Versorgungsübergängen während einer hochkritischen Phase führen [49, 50].

Der Erfolg des Frühinterventionsmodells bei psychotischen Störungen diente als „proof of principle“ für Früherkennung und -intervention insgesamt und war globaler Auslöser für signifikante Investitionen in diesem Bereich – nicht nur für psychotische Erkrankungen, sondern für die ganze Bandbreite von psychischen Erkrankungen. Diese Initiativen konzentrieren sich auf junge Menschen zwischen 12 und 25 Jahren (die unbegründete Grenze zum 18. Lebensjahr wird vermieden). Sie bieten ein breites Spektrum der Gesundheitsversorgung (im Sinne eines One-Stop-Shops) und einen barrierefreien, einfachen Zugang für junge Menschen („soft entry“) als besonders wichtigen Bestandteil [41, 51, 52].

Im Jahr 2004 begann die australische Regierung das „Headspace-Modell“ zu finanzieren, ein niederschwelliges Programm zur psychischen Gesundheitsförderung und Prävention für junge Menschen, welches innerhalb weniger Jahre zu 136 Zentren anwuchs und jährlich von 130.000 jungen Australiern genutzt wird [52]. Dem Headspace-Modell folgten schnell ähnliche Initiativen in Irland (Jigsaw; [53]) und Kanada (ACCESS Open Minds; [54]). Dänemark, die Niederlande, Israel und Island haben kürzlich ein integriertes, Headspace-informiertes primäres Versorgungsprogramm für junge Menschen eingeführt. Ähnliche und parallele Entwicklungen sind in Frankreich zu verzeichnen (Maison des Adolescents; [55, 56]). Diese neuen Versorgungsangebote ermöglichen eine breite, transdiagnostische Herangehensweise, die systematische Untersuchung früher Stadien psychischer Erkrankungen sowie die Untersuchung neuer Interventions- und Präventionsstrategien. Doch diese Anstrengungen im Bereich der Früherkennung sind nicht ohne Kritik geblieben.

Die Kritik an Hochrisikostrategien bezieht sich im Wesentlichen darauf, dass hier das Interventionsziel insbesondere die Veränderung von individuellen Risiken ist, die zum einen zu einer (Selbst‑)Stigmatisierung derjenigen Individuen führen können, die eine Exposition gegenüber diesen Risiken erfahren und de facto als Hochrisikoindividuen klassifiziert werden [57]. Zum anderen kann der Fokus auf Risiken auf individueller Ebene dazu führen, dass deren Reduktion nicht die Verteilung von neu auftretenden Risiken in der Gesellschaft beeinflusst, da nicht die Bedingungen adressiert und modifiziert werden, unter denen das hohe Risiko entsteht, und somit immer neue Individuen eine hohe Risikoexposition erfahren [58]. Obwohl bei Hochrisikostrategien der Nutzen für das kleine Segment der Hochrisikopopulation hoch sein kann, bleibt der Nutzen – gemäß dem Präventionsparadox von Rose [7, 8] – für die gesamte Population und die Veränderung der Risikoverteilung insgesamt begrenzt.

### Populationsstrategie: Verschiebung der Verteilung von Risiken und Gesundheit auf Populationsebene

Ein wichtiger Bezugspunkt für das Fach Public Mental Health ist die wegweisende Populationsstrategie von Geoffrey Rose, die die Verteilung von Risiken und Gesundheit der *gesamten* Population in den Blick nimmt, verbunden mit der Erkenntnis, dass bei Gesundheitsproblemen, die in der Population kontinuierlich verteilt sind, bereits eine geringe Verschiebung der durchschnittlichen Gesundheit auf Populationsebene (d. h. des Populationsmittelwerts) einen erheblichen Einfluss auf die Prävalenz des Gesundheitsproblems haben kann [7]. Rose hatte dies bereits in den 1990er-Jahren für gesundheitsbezogene Charakteristiken wie Blutdruck, Body-Mass-Index und Alkoholkonsum untersucht und bspw. in der Intersalt-Studie [59] gezeigt, dass der mittlere Alkoholkonsum sehr eng mit der Prävalenz des problematischen Trinkens korreliert. Zudem war eine nur geringfügige Reduktion im mittleren Alkoholkonsum (von nur 15 ml pro Woche) mit einer erheblichen Reduktion in der Prävalenz des problematischen Trinkens assoziiert [59].

Um dieses Shifting the Curve zu illustrieren, verwendete Rose für die Verteilung von psychischer Gesundheit auf Populationsebene die Metapher eines Eisbergs, um aufzuzeigen, dass die Spitze des Eisbergs als der für uns sichtbare Teil – also die gemessene Prävalenz von behandelten psychischen Gesundheitsproblemen – eine Funktion seiner gesamten Masse – also des Populationsmittelwerts – ist [7]. Bei der Ausrichtung von Surveillance, Monitoring und evidenzbasierter Planung der Gesundheitsversorgung ist es in diesem Sinne zentral für Public Mental Health, nicht nur den sichtbaren Teil, sondern die gesamte Masse des Eisbergs, also die Verteilung der psychischen Gesundheit auf Populationsebene, im Blick zu haben [3]. Dies lässt die Validierung und Kalibrierung dimensionaler Modelle sowie deren Maße der zentralen Tendenz in Bezug auf evidenzbasierte Risikoschwellen umso wichtiger erscheinen. Mittlerweile ist der Zusammenhang zwischen Populationsmittelwert und Prävalenz tatsächlich auf Bevölkerungsebene sowie deren Verteilung entlang eines Kontinuums gut dokumentiert [28–33].

Allerdings bleiben wichtige kritische Fragen hinsichtlich der Populationsstrategie von Rose [7] unbeantwortet. So hat sich die damit befasste internationale Forschung bislang ausschließlich auf den Zusammenhang zwischen Populationsmittelwert und *Prävalenz* von psychischen Erkrankungen, nicht jedoch auf deren *Inzidenz* bezogen. Wenn wir jedoch den Public-Health-Impact der öffentlichen psychischen Gesundheitsversorgung – d. h. von Gesundheitsförderung, Prävention, Frühintervention, psychiatrischer und psychotherapeutischer Versorgung – auf Populationsebene untersuchen wollen, bedarf es letztlich auch der Evidenz über die mit der Veränderung des Populationsmittelwerts assoziierte Reduktion der Inzidenz. Damit wäre eine Grundlage geschaffen, den Public-Health-Impact der Gesundheitsförderung, Prävention, Früherkennung und Frühintervention direkt messbar zu machen [3]. Während in Deutschland die letzte Studie zur Inzidenz von psychotischen und anderen schweren psychischen Störungen mehr als 20 Jahre zurückliegt [60–65] und dadurch ein riesiger blinder Fleck in der psychiatrischen Epidemiologie entstanden ist, gibt es aus internationalen Studien zumindest starke und konsistente Evidenz zur Inzidenz dieser Störungen in Abhängigkeit von Urbanisierungsgrad sowie Ethnizität [60–63]. Aber auch international bleibt die Untersuchung des zeitlichen Zusammenhangs der Inzidenz mit dem Populationsmittelwert eine drängende grundsätzliche Frage im Bereich der Public Mental Health [3].

Zudem stellt sich die nicht minder schwer zu beantwortende Frage, ob es grundsätzlich möglich ist, eine mittlere Verbesserung der psychischen Gesundheit auf Populationsebene zu bewirken [9]. Angeführt werden kann hier die zunehmend stärkere Evidenzlage zur Wirksamkeit von Maßnahmen der Public Mental Health bspw. im Bereich der psychischen Gesundheitsförderung und Prävention in Schulprogrammen (insb. multimodal auf die gesamte Schule ausgerichtete Programme; [66–69]) oder Elterntrainingsprogrammen (z. B. prä- und perinatale oder auf die Verbesserung der emotionalen und sozialen Entwicklung ausgerichtete Programme; [70–72]). Um jedoch präzise und robuste Evidenz auf Populationsebene zu generieren, bedarf es zunächst einer empirisch validierten Taxonomie und kontinuierlichen Erfassung relevanter Parameter der populationsbasierten psychischen Gesundheit (z. B. Inzidenz, Maße der zentralen Tendenz, Krankheitslast, psychische Beeinträchtigung).

Ein weiterer grundlegender Kritikpunkt an der Populationsstrategie von Geoffrey Rose bezieht sich auf die erhöhte Variation in der Risikoverteilung, die zur Folge hat, dass diejenigen, die zuvor (d. h. zur Baseline, vor Durchführung der populationsbasierten Intervention) eine geringere Risikoexposition erfahren haben, mehr von Populationsstrategien profitieren als diejenigen, die vormals eine höhere Exposition gegenüber Risiken erfahren haben [39]. Kurzgefasst bezieht sich die Kritik darauf, dass der Gesamteffekt von Populationsstrategien auf die Risikoreduktion verschleiert, dass dieser Effekt maßgeblich von der Risikoexposition zur Baseline abhängen kann (d. h. durch diese konfundiert ist) und somit unbeabsichtigt zu sozialen und gesundheitlichen Ungleichheiten beiträgt [39]. Während es für dieses Ungleichheitsparadox (wie es Frohlich und Potvin [39] benannt haben) als unbeabsichtigten Effekt von Populationsstrategien bereits seit Längerem empirische Befunde bspw. aus Anti-Rauch-Kampagnen [73, 74] oder dem Screening auf Brust- und Gebärmutterhalskrebs [75] gibt, von denen Menschen mit höherem sozioökonomischen Status mehr profitieren als andere, bleibt die Evidenz für den Bereich der psychischen Gesundheit begrenzt.

### Strategien zur Verringerung gesundheitlicher Ungleichheiten bei vulnerablen Populationen

Die aus der Kritik an der Populationsstrategie erwachsene Erkenntnis des Ungleichheitsparadoxes lenkt die Aufmerksamkeit auf fundamentale Ursachen sozialer und gesundheitlicher Ungleichheiten (nach Link und Phelan [76, 77]), die zu unterschiedlichen Risikoverteilungen für Subpopulationen in Abhängigkeit von deren Position in der Sozialstruktur führen. Das Ziel populationsbasierter Interventionen verschiebt sich somit in der Konsequenz auf vulnerable (Sub)Populationen, die aufgrund geteilter sozialer Charakteristiken (z. B. Ethnizität, Bildungsstand, sozioökonomischer Status) ein höheres Risiko einer Risikoexposition aufweisen und deshalb systematische soziale und gesundheitliche Ungleichheiten erfahren. Diese gilt es – gemäß einem vulnerablen Populationsansatz [39] – zu reduzieren.

Der Begriff vulnerabler Populationen bezieht sich dabei auf Gruppen, die aufgrund ihrer Position in den sozialen Schichten eine Exposition gegenüber kontextuellen Bedingungen erfahren, die sie vom Rest der Population abgrenzen und den Mittelwert ihrer Risikoexpositionsverteilung höher ausfallen lassen. Im Unterschied zur Hochrisikostrategie erfahren in diesem Ansatz vulnerable Populationen aufgrund gemeinsamer sozialer Charakteristiken eine höhere durchschnittliche Exposition gegenüber zahlreichen Risikofaktoren, aber nicht jedes Mitglied einer vulnerablen Population erfüllt die Kriterien (z. B. eines CHARMS), durch die es einer Hochrisikopopulation zuzuordnen wäre [39]. Das zentrale definitorische Element sind die geteilten sozialen Charakteristiken, durch die Mitglieder einer vulnerablen Population ein durchschnittlich höheres Risiko einer Risikoexposition erfahren. Ziele von populationsbasierten Interventionen werden somit fundamentale Ursachen sozialer und gesundheitlicher Ungleichheiten, die Vulnerabilität in den Lebenswelten vulnerabler Populationen entstehen lassen.

Da die Position einer Person in der Verteilung von Gesundheit von allen vorhergehenden – auch nicht gesundheitsbezogenen – Erfahrungen abhängt [78], gilt hier ein besonderes Augenmerk auf kumulative Effekte der (unbeabsichtigten) Konzentration und Intersektionalität (d. h. Überschneidung/Gleichzeitigkeit) von Risiken. Während es mittlerweile Evidenz über die komplexe Wirkweise sozialer und Umweltrisiken und deren Intersektionalität (z. B. die Überschneidung verschiedener Diskriminierungskategorien gegenüber einer Person) in der Entstehung psychischer Erkrankungen über die Lebensspanne gibt [79–83], müssen Interventionen, die diese kumulativen und multiplen Risiken sowie fundamentale Ursachen sozialer und gesundheitlicher Ungleichheiten von vulnerablen Populationen in den Blick nehmen, in der Public Mental Health noch stärkere Aufmerksamkeit erfahren.

### Inklusion vulnerabler Populationen als zentrale Herausforderung der Public Mental Health

Die unbeabsichtigten Effekte einer Verstärkung und Verstetigung von sozialen und gesundheitlichen Ungleichheiten durch Populationsstrategien müssen eine zentrale Rolle bei der Entwicklung, Evaluation und Implementierung von neuen Interventionen zur Verbesserung der populationsbasierten Gesundheit spielen. Sie lassen aktuelle Konzeptualisierungen von individuellen, sozialen und strukturellen Determinanten der Public Mental Health umso wichtiger erscheinen. Die Untersuchung und die Reduktion dieser unbeabsichtigten Effekte müssen im Mittelpunkt stehen, um vulnerable Populationen mit verschiedenartigen sozialen und ethnischen Hintergründen nicht zu benachteiligen, sondern bei der Verbesserung der populationsbasierten Gesundheit, dem Shifting the Curve, zentral zu berücksichtigen und zu inkludieren.

Ein wichtiger Ansatzpunkt dafür ist die Verwendung partizipativer Forschungsmethoden, die die Belange von vulnerablen Populationen durch die Beteiligung an der Entwicklung von gesundheitsfördernden und präventiven Maßnahmen sowie an deren Evaluation (durch Co-Design und Co-Production) zentral berücksichtigen. Aber auch Maßnahmen, deren Ziel die Modifikation von kumulativen Risiken und deren Intersektionalität bei vulnerablen Populationen ist, stellen einen wichtigen Ansatzpunkt dar. Dies gilt auch für eine genuin intersektorale Vorgehensweise, die die fundamentalen Ursachen gesundheitlicher Ungleichheiten nicht auf den Sektor der Gesundheitsversorgung beschränkt betrachtet, sondern mit anderen Sektoren bei der Verringerung dieser Ungleichheiten zusammenarbeitet. Eng damit verknüpft ist ein Verständnis von psychischer Gesundheit, das im Sinne eines Mental-Health-in-All-Policies(MHiAP)-Ansatzes [84] das Ziel verfolgt, Auswirkungen von politischen Entscheidungen auf psychische Gesundheit in allen gesellschaftlichen Handlungsfeldern als gemeinschaftliche Aufgabe zu berücksichtigen, um nicht dem „Ungleichheitsparadox“ bei der Entwicklung neuer populationsbasierter Interventionen Vorschub zu leisten. Verhältnispräventive Maßnahmen, deren Ziel eine Veränderung der Lebenswelten ist, die das Risiko der Exposition gegenüber Risikofaktoren von vulnerablen Populationen erhöhen, müssen dabei ebenfalls eine wichtige Rolle spielen, um den fundamentalen Ursachen von sozialen und gesundheitlichen Ungleichheiten im Alltag zu begegnen.

Zusammengefasst bleibt es somit eine übergeordnete Herausforderung der Public Mental Health, die fundamentalen Ursachen von gesundheitlichen und sozialen Ungleichheiten beim Shifting the Curve zu verringern, indem in allen Bereichen der öffentlichen psychischen Gesundheitsversorgung der Einfluss von Faktoren begrenzt wird, die mit einem erhöhten Risiko der Risikoexposition bei vulnerablen Populationen einhergehen. Anstrengungen in Politik, Forschung und Praxis, die diese Herausforderung in allen gesellschaftlichen Handlungsfeldern annehmen, lassen eine tatsächliche Verbesserung der psychischen Gesundheit der gesamten Bevölkerung wahrscheinlicher werden.
